# Preferential Feeding and Occupation of Sunlit Leaves Favors Defense Response and Development in the Flea Beetle, *Altica brevicollis coryletorum* – A Pest of Corylus avellana

**DOI:** 10.1371/journal.pone.0126072

**Published:** 2015-04-30

**Authors:** Adrian Łukowski, Marian J. Giertych, Marcin Zadworny, Joanna Mucha, Piotr Karolewski

**Affiliations:** 1 Institute of Dendrology, Polish Academy of Sciences, Kórnik, Poland; 2 Department of Forest Protection, Faculty of Forestry, Poznań University of Life Sciences, Poznań, Poland; 3 Faculty of Biological Sciences, University of Zielona Góra, Zielona Góra, Poland; Henan Agricultural Univerisity, CHINA

## Abstract

The monophagous beetle, *Altica brevicollis coryletorum*, is a major leaf pest of *Corylus avellana* (common hazel). In contrast to majority of the other studied species of shrubs, sunlit leaves are grazed to a much greater extent than shaded leaves. Since the observation of a link between leaf irradiance level and *A*. *brevicollis* feeding is unique, we hypothesized that feeding preference of this beetle species is related to the speed needed to escape threats i.e. faster jumping. We also hypothesized that sunlit leaves are more nutritious and easier to consume than the leaves of shaded shrubs. Results indicated that beetle mass was greater in beetles occupying sunlit leaves, which is consistent with our second hypothesis. The study also confirmed under laboratory conditions, that larvae, pupae and beetles that were fed full-light (100% of full light) leaves were significantly heavier than those fed with shaded leaves (15% of full light). In the high irradiance conditions (higher temperature) duration of larval development is also reduced. Further results indicated that neither the concentration of soluble phenols, leaf toughness, or the number of trichomes could explain the insect’s preference for sunlit leaves. Notably, measurements of jump length of beetles of this species, both in the field and under laboratory conditions, indicated that the defense pattern related to jumping was associated with light conditions. The jump length of beetles in the sun was significantly higher than in the shade. Additionally, in laboratory tests, beetle defense (jumping) was more strongly affected by temperature (15, 25, or 35°C for 24h) than by leaf type. The effect of sunlit, higher nutrient leaves (greater level of non-structural carbohydrates) on defense (jumping) appears to be indirect, having a positive effect on insect mass in all developmental stages.

## Introduction

Temperate forests are characterized by a diverse understory, including large numbers of herbaceous, shrub, and tree species [1,2]. Native species of the Betulaceae, including the genus *Corylus* L., play an important role in the understory of European forests [3,4]. *C*. *avellana* L. (common hazel) is widely distributed and serves as a food source for a variety of herbivores, particularly herbivorous insects [5,6]. One of the dominant folivores of *C*. *avellana* is the flea beetle, *Altica brevicollis coryletorum* [7,8] which is a typical monophagous insect. In literature there is lack of information about the occurrence of this species on other plant species than *C*. *avellana*. Beetles of this monophagous species are very numerous on hazel shrubs and cause substantial defoliation [9]. Since hazelnuts have a high nutritional value and a significant role in human diet [10], studies to investigate the biology and ecology of this insect pest are essential [6].

Among its many beneficial functions, the understory serves as a rich source of food for insect herbivores [11,12]. The high level of insect diversity in the understory, and the high abundance of folivores, result from the fact that understory plants grow mostly in shade [13]. Earlier studies indicated that shade-grown leaves of plants are more damaged by insects than those growing in full light because of the lower level of defense compounds in the shade leaves [14,15]. Leaves of shaded plants tend to be thinner, softer and less tough [16,17] and their surface has fewer structures that impede insect movement and grazing [18,19]. Leaf structure and chemistry jointly determine food quality and food preferences for folivores [20]. Insect feeding sites may also be directly determined by light conditions and, consequently, temperature. This relationship is due to the amount of energy used by insects and also with insect tolerance to extremely high temperatures [21].

**Fig 1:**
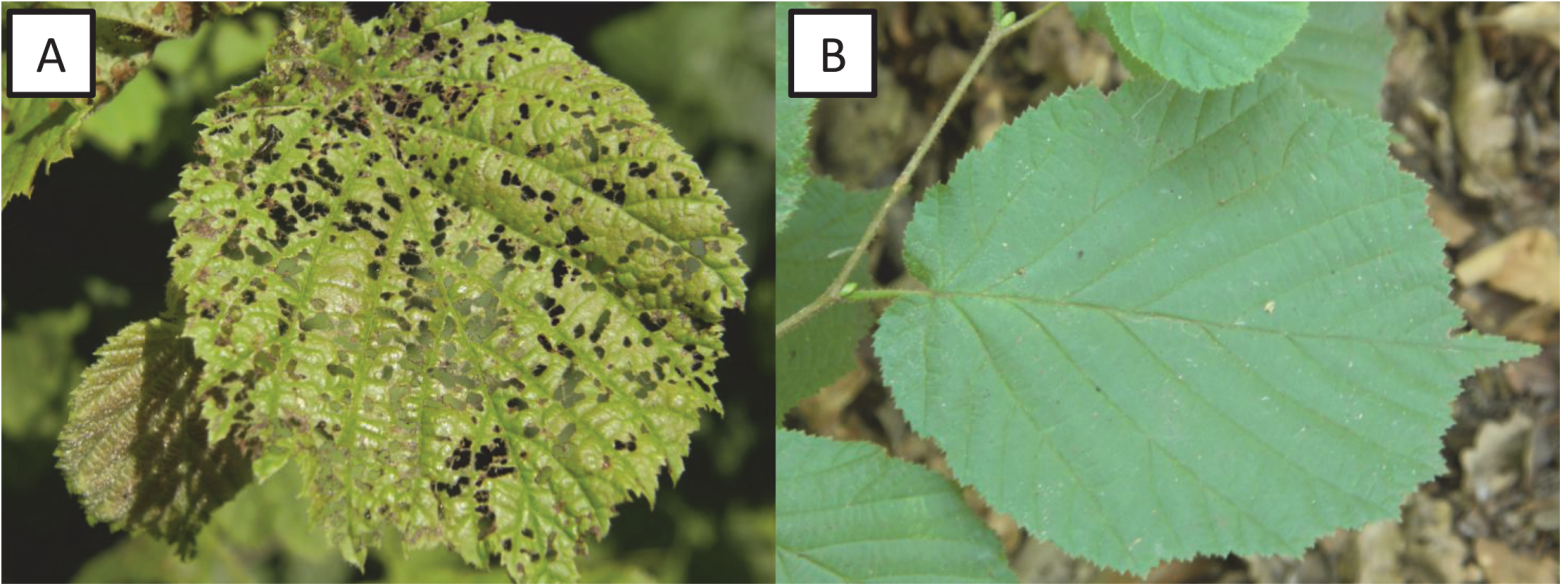
Occupation and feeding of the flea beetle, *Altica brevicollis coryletorum* on sunlit (A) and shaded (B) leaves of common hazel, *Corylus avellana*.

The combined factors mentioned above results in less folivore damage to sunlit leaves than shaded leaves in most species of woody plants. In contrast to this general premise, our previous research on *C*. *avellana* [9] and recent observations (Fig 1) show an opposite pattern, i.e. a much higher level of feeding and defoliation of sunlit rather than shaded common-hazel shrubs. Observations of the high degree of sunlit leaf damage also corresponded to a higher abundance of larvae and adults of *A*. *brevicollis coryletorum* [9]. Therefore, a major objective of the current research was to identify the reasons for the preference of specialist species *A*. *brevicollis coryletorum* for shrubs growing in full light. Our first hypothesis was that *A*. *brevicollis coryletorum* prefer sunlit leaves of *C*. *avellana* due to a greater nutritional value and primarily a higher content of non-structural carbohydrates. The relationship between light and non-structural carbohydrate content was identified in our earlier study [9]. Our second hypothesis was that the less favorable structural properties of sunlit leaves for insect consumption is not a limiting factor for the feeding of *A*. *brevicollis coryletorum* on sunlit leaves of *C*. *avellana*. The current study also addressed and verified a third hypothesis, namely, that the major factor affecting the feeding preferences of *A*. *brevicollis coryletorum* is the light conditions present for plant growth, which influences the defense response of the beetles against predators. Jumping was the defense response to predators focused on in the present study since it is a primary defense mechanism characteristic of adult insects of this species. While jumping is a frequent mode of locomotion in *A*. *brevicollis coryletorum*, it primarily represents a way to quickly escape danger [22,23]. In this context, the present study attempted to determine which factor has the greatest impact on the jumping defense response: the higher nutritional value of sunlit leaves or the higher temperature of sunlit leaves.

## Materials and Methods

The study was conducted on the native leaf beetle, *Altica brevicollis coryletorum* (Coleoptera, Chrysomelidae, [24]). The studied species is one of the major pests of common hazel (*Corylus avellana* L.), usually chemically controlled in plantations in Poland. The genus *Altica* is the type genus of the Alticinae, generally referred to as flea beetles, the largest subfamily of leaf beetles (Chrysomelidae) [25]. All larval stages, pupae, and adult insects were used in the laboratory experiments while only adult insects were used in the field research. This study was carried out in strict accordance with the ethical standards in entomological research.

Field and laboratory experiments were carried out on shrubs and seedlings of common hazel, a frequent understory species. Common hazel is a tree, or more typically a shrub, that may grow up to 6 m high. Its leaves are rounded, 6–12 cm long and wide, softly hairy on both surfaces, with a double-serrate margin [26]. The collection of seeds (in this case, hazel nuts) is in Poland allowed without additional permission.

### Field research

The study was performed in Poland at three locations: the Experimental Forest “Zwierzyniec” in Kórnik (52°14’N, 17°05’E), Tulce (Kobylepole Forest, Babki Forest District; 52°36’N, 17°06’E), and the Palędzie (Palędzie Forest, Konstantynowo Forest District; 52°23’N, 16°40’E). All field works in Tulce and Palędzie were agreed with the field managers of the State Forests while in the Experimental Forest “Zwierzyniec” in Kórnik all experiments were consulted with the Director of Institute of Dendrology. *C*. *avellana* shrubs 3–5 m high growing in either full light or shaded conditions (ca. 15–30% of full light when measured in June, with the LAI-2200 plant canopy analyzer, LI-COR Environmental, USA) were used in the study. The shrubs were growing under a canopy of *Pinus sylvestris* L. with an admixture of *Quercus robur* L., *Fagus sylvatica* L., *Carpinus betulus* L. and *Ulmus laevis* Pall. On each site (*n* = 3) 10 shrubs (*n* = 10) per light variant (*n* = 2; sunlit and shaded shrubs) were selected, for a total of 60 shrubs.

Beetles were caught in mid-August 2013 (current-year generation of beetles) and in mid-May 2014 (after the winter diapause; 1-year-old beetles) in order to measure adult insect mass. The number of captured insects was approximately15-30 individuals/shrub for each variable of the experiment. When the number of insects on a shrub was too low (<10), which was the case for shaded shrubs, they were supplemented with insects from a neighboring shrub. The captured beetles were killed with ethyl acetate, and their mass and sex were determined about 18 h after their death.

### Laboratory experiment

Seeds of common hazel, obtained from the Palędzie Forest (the same as described above) were sown, after stratification (Laboratory of Seed Biology (Institute of Dendrology, Kórnik), in pots (1.5 dm^3^ each) during mid-April 2007. The pots were filled with forest soil (collected in a mature oak/pine forest) that was mixed with neutralized peat (1:1) and a slow-release N-P-K fertilizer Osmocote (2 kg m^-3^). Seedlings were divided into 2 groups and grown under 15% or 100% full light. All shrubs were watered as necessary.

Separate larvae of *A*. *brevicollis coryletorum* were collected in Kórnik, at the earliest possible stage of their development (June 8, 2013), from shrubs growing in full light or from shaded shrubs. A total of 40 individuals were collected from each type of shrub. The larvae from sunlit shrubs were fed leaves from 6-year-old seedlings growing in full sunlight (100%), while the larvae from shaded shrubs were fed leaves of seedlings grown under a shade cloth (15% of natural light). Each larva was reared singly in Petri dish in the laboratory at room temperature. After each leaf was weighed, the petiole of each leaf was placed in a punctured lid of an Eppendorf tube filled with water to prevent leaves from withering and drying out. Leaves were replaced every 2 days. The uneaten remnants of leaves were weighed after drying at 65°C for 48 h.

Various parameters of insect growth and development were used as indicators of insect performance and growth [27]. Relative growth rate (RGR) was calculated every two days by measuring larval mass. The duration of larval development (DD), i.e. the time from the beginning of the experiment to pupation, as well as pupal mass (PM), was also recorded. Beetles were killed with ethyl acetate two days after eclosion and their fresh body mass and sex determined about 18 h after their death. RGR was calculated using the formula: RGR = (M_t_–M_0_)/(T_t-0_×M_0_), where M_0_ and M_t_ are initial and final larval mass (in mg), respectively, and T_t-0_ is the time between the initial and final measurement (in days). The efficiency of conversion of ingested food (ECI) was calculated based on the larval mass and total mass of food eaten (TFE), using the following formula: ECI = (final larval mass/TFE) × 100%. TFE (as dry leaf mass) was calculated by subtracting the dry mass of leaf remnants from the estimated dry mass of the leaf before placing it in a Petri dish. The dry mass of the fresh leaves placed on Petri dishes was estimated by comparing the fresh mass of a part of leaves from each light variant with their dry mass after desiccation at 65°C in order to obtain a fresh/dry mass ratio. Greater details on the methods used in the present study can be found in our earlier reports [28–30]. All masses were measured with an analytical balance (Sartorius CP225D; ± 0.01 mg).

### Leaf toughness

The toughness as a mechanical parameter of plant protection against herbivory is usually using in studies connected with light conditions of leaf growth [17]. Toughness of leaves measured with penetrometer is expressed by punch strength (gf) needed to pass a leaf lamina by rod of given the tip area (mm^-2^). Leaf toughness was measured throughout the laboratory experiment (May 27; June 17; July 8; July 29), using a penetrometer with tip size 3.5 mm (FHT 801; Equipment Depot, Fotronic, Melrose, MA, USA). Leaf toughness (gf mm^-2^) was measured in 80 undamaged leaves (2 light conditions × 4 dates) by performing two measurements between the lateral veins, on the left and the right half of the leaf.

### Chemical analysis

The chemical composition (concentration of nitrogen, non-structural carbohydrates, soluble phenolic compounds, and condensed tannins) of leaves of shrubs of *C*. *avellana* growing in either the shade or in full light were determined in our earlier study [9]. In the present study, the concentration of these constituents were only measured in leaves of the seedlings of *C*. *avellana* used in the laboratory experiment. Leaves from three seedlings growing in full light (100%), and three seedlings growing in reduced light (15% of full light), were measured on four dates (May 27; June 17; July 8; and July 29, 2013).

The analyses utilized powdered leaf tissue, obtained from leaves previously dried at either 40°C for the condensed tannins, or at 65°C for the other compounds. The concentration of total soluble phenols was measured colorimetrically using Folin and Ciocalteu’s Phenol Reagent, while condensed (catechol) tannins were measured using a color reaction with vanillin in an acid medium. Results of phenol measurements were expressed per μM chlorogenic acid in g^-1^ d.m., while condensed tannins were converted into μM catechin g^-1^ d.m.

Total non-structural carbohydrates (i.e. soluble carbohydrates and starch) were determined colorimetrically. Soluble carbohydrates were assayed in methanol-chloroform-water extracts. The total amount of soluble carbohydrates and starch (using glucose as a standard) was expressed as percentage of dry mass (% d.m.). Nitrogen content (% d.m.) was determined using an Elemental Combustion System CHNS-O 4010 analyzer (Costech Instruments, Italy/USA; http://www.costechanalytical.com). Seedling leaf analyses were performed in the same manner as described in detail in our previous report [9].

### Histochemical analysis and anatomical measurements

Fresh, hand-made sections were used for the histochemical analyses. The distribution of phenols within trichomes of *C*. *avellana* was examined using toluidine blue [31]. Each section was observed at 10, 20 and 40 × magnification with an Axioskop 20 microscope. The proportion of trichomes with phenolic compounds staining, relative to the total number of observed trichomes, was then calculated.

For anatomical measurements, 1-mm^2^ sections of *C*. *avellana* leaves were fixed with a mixture of 4% (v/v) glutaraldehyde and 4% paraformaldehyde (1:1; w/v; pH 6.8; Polysciences, Warrington, USA) in 0.5 M cacodylate buffer at pH 7.2 for 24 h. The specimens were then washed in distilled water, dehydrated in a graded ethanol series followed by a graded acetone series, and dried in a critical point dryer (Balzers CPD-030) using CO_2_ as a transition fluid. Dried sections were mounted on clean aluminum stubs with double-sided adhesive graphite tabs. Mounted specimens were coated with gold (12–15 nm thick), using a Balzers SPD-050 sputter coater. Sections were examined on an EVO40 scanning electron microscope (Carl Zeiss, Germany) and digitally photographed.

### Jump length

Jump lengths of current-year beetles of *A*. *brevicollis coryletorum* were measured on August 6, 2013 in order to determine the effect of the light conditions of shrubs on the defense reaction of the flea beetle. Measurements were performed on flea beetles immediately after taking them from sunlit (*n* = 120 insects) or from shaded (*n* = 80) *C*. *avellana* shrubs in Tulce. Each beetle was placed in a funnel directing it to the center of a target with a measurement scale. This enabled measurements jump length to the nearest 0.5 cm. The first jump length of each beetle was used.

The direct effect of temperature or its indirect effect (based on the quality of leaves as food, which was determined by local light conditions) on the defense reaction of *A*. *brevicollis coryletorum* was also assessed under laboratory conditions. Adult beetles were collected on August 12, 2014 from sunlit and shaded shrubs growing at the Tulce site and fed for 24 h in the laboratory with fresh leaves of the shrubs from which they had been caught. The insects were then individually placed in 125-ml boxes with fresh leaves from corresponding light conditions, and placed at one of three temperatures (15, 25 or 35°C). Approximately 100 beetles (*n*≈100) per light variant (*n* = 2) and temperature variant (*n* = 3), were used, representing a total of about 600 beetles. After 24 h, each beetle was immediately placed in a funnel directing it to the center of the target and its jump length was measured. After measuring the jumps, the insects were killed and their mass and sex determined.

### Statistical analyses

Analysis of covariance (ANCOVA) was used to assess the influence of light conditions and sex on insect masses, efficiency of conversion of ingested food (ECI), duration of larval development (DD), total mass of food eaten (TFE), and relative growth rate (RGR) for an initial period of rectilinear changes, i.e. in this case after ten days. The initial mass was used as a covariate. ANCOVA was also used to assess the influence of light conditions and sex in the field experiment. Additionally, ANCOVA was used to assess the influence of food quality (leaf type), temperature and sex on beetle jump length in the controlled experiment, where beetle body mass was considered as a covariate. Restricted maximum likelihood analysis of variance (REML ANOVA) was used to assess the influence of light conditions, sex and age on beetle mass and sex ratio (F/M), where the research site was treated as a random factor. For results of chemical analysis, ANOVA was used on the data obtained from the chemical analyses in order to assess the influence of light conditions and date.

**Fig 2:**
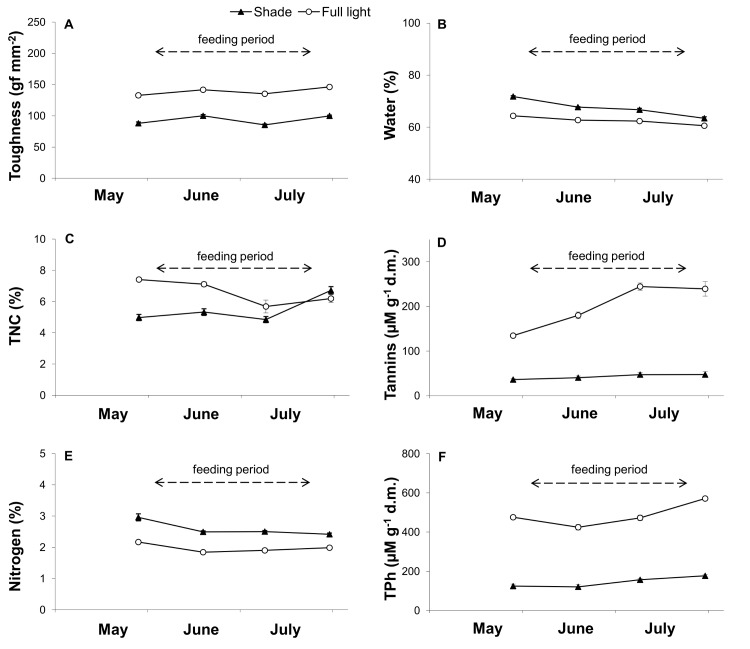
Effect of light conditions (100% sunlight vs. 15% sunlight) on various leaf parameters in seedlings of *Corylus avellana* during the larval feeding period. **Leaf toughness (A), water content (B), total non-structural carbohydrates (C), condensed tannins (D), nitrogen (E) and total soluble phenols (TPh) (F).** A one-way ANOVA was used to determine the significance of differences in leaf toughness (A) for light (*P*<0.0001), date (*P*<0.0001) and light × date (*P* = 0.2784); in water content (B) for light (*P*<0.0001), date (*P*<0.0001) and light × date (*P*<0.0001); in total non-structural carbohydrates (C) for light (*P*<0.0001), date (*P* = 0.0004) and light × date (*P*<0.0001); in condensed tannins (D) for light (*P*<0.0001), date (*P*<0.0001) and light × date (*P*<0.0001); in nitrogen (E) for light (*P*<0.0001), date (*P*<0.0001) and light × date (*P* = 0.0237); in total soluble phenols (F) for light (*P*<0.0001), date (*P*<0.0001) and light × date (*P*<0.0030). Error bars denote standard error of the mean.

**Fig 3:**
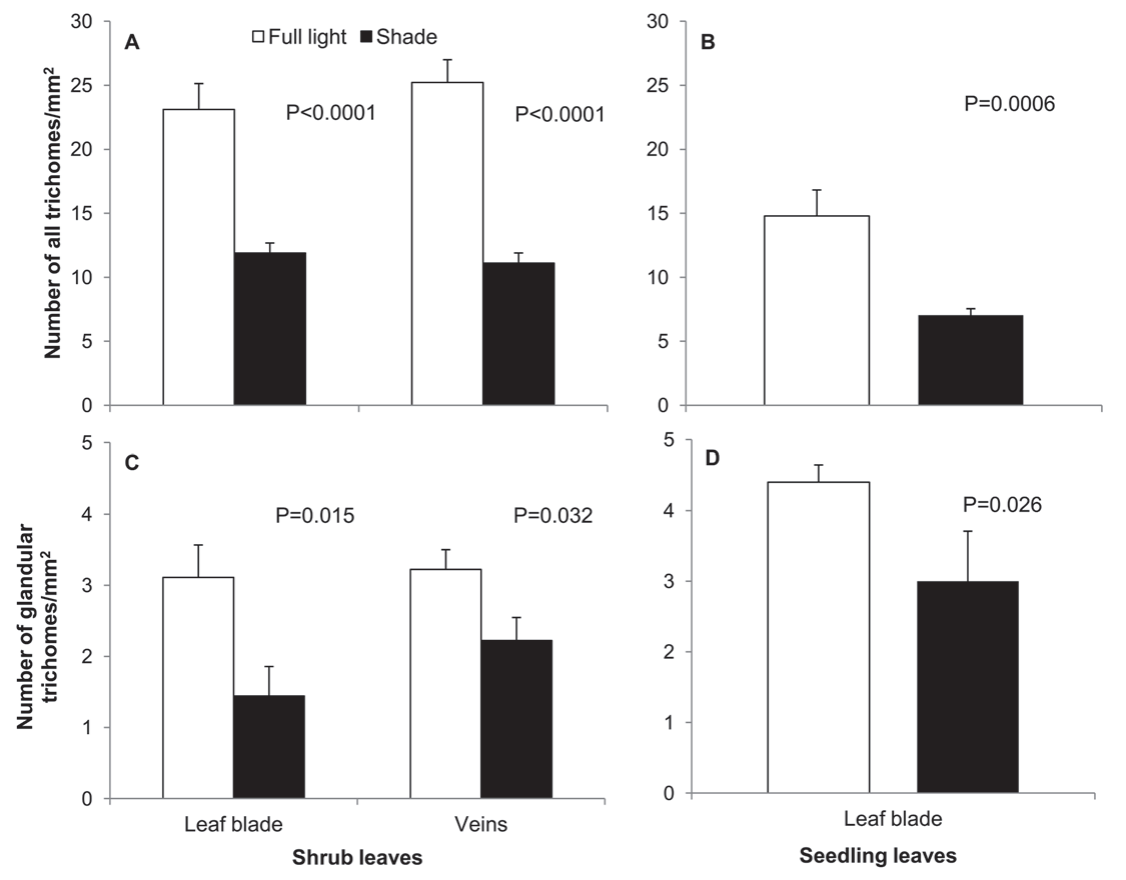
Density (number of trichomes\ mm^2^ leaf blade) of all trichomes and glandular trichomes present on the leaf blade or along leaf veins, respectively, of leaves of *Corylus avellana* shrubs growing under field conditions (A, C) or seedlings growing und controlled, laboratory conditions (B, D). Error bars denote standard error of the mean.

All statistical analyses were conducted using JMP 8 (SAS Institute, Cary, NC, USA) software. For data expressed as percentages (total non-structural carbohydrates and N content), the Bliss arcsin formula was used [32]. Tukey’s HSD test was employed to assess the significance of differences between treatments. Error bars denote the standard error of the mean (SEM; Figs 2 and 3).

## Results

### Field research

A significant effect of light conditions on adult insect mass of *A*. *brevicollis coryletorum* was observed in the field studies (Table 1). The beetles feeding on shrubs growing in full light were 22.9% heavier than those found in the shade. Moreover, females were 54.7% heavier than males. While beetles of both sexes feeding on sunlit shrubs were heavier, the light × sex interaction was also significant, as light conditions had a greater influence on the mass of females than males. Adult insect mass was the highest for females feeding in full light, while the lowest was in males found on shaded shrubs (Tukey’s HSD test; *P*<0.05).

**Table 1:** Mean values (and standard error) of *Altica brevicollis coryletorum* female and male beetle mass in relation to insect age and light conditions, and a summary of the ANOVA results.

Treatment		Number of individuals		Beetle mass (mg)	
Light	Age	Female	Male	Female	Male
Full	current-year	281	297	6.76 (0.12)	4.99 (0.10)
	1-year-old	287	145	8.78 (0.10)	5.24 (0.07)
Shade	current-year	205	211	5.48 (0.10)	3.98 (0.10)
	1-year-old	213	171	7.36 (0.13)	4.45 (0.08)
ANOVA		d.f.	Error	*F*	*P*
Light		1	1787	216.7148	**<0.0001**
Age		1	1788	206.3425	**<0.0001**
Sex		1	1786	1001.550	**<0.0001**
Light × age		1	1788	2.3188	0.1280
Light × sex		1	1787	20.2279	**<0.0001**
Light × site		2	1787	9.9364	**<0.0001**
Age × sex		1	1786	116.0142	**<0.0001**
Age × site		2	1787	159.3946	**<0.0001**
Sex × site		2	1788	8.8564	**0.0001**
Light × age × sex		1	1787	6.9937	**0.0083**
Light × age × site		2	1787	2.8436	0.0585
Light × sex × site		2	1787	0.8505	0.4274
Age × sex × site		2	1788	8.4158	**0.0002**
Light × age × sex × site		2	1787	0.3138	0.7307

Data was obtained from beetles at three different research sites (Kórnik, Palędzie, and Tulce).

Insect age also significantly affected adult mass. One-year-old beetles feeding in summer were 27.9% heavier than current-generation beetles collected in summer. This can be partially explained by the significant age × sex interaction, as in spring, females were the heaviest (observations indicated that they were already fertilized), while male mass did not differ significantly with beetle age (Tukey’s HSD test; P<0.05).

Insect age also significantly affected the sex ratio (F/M) at the time of beetle collection (Table 2). The F/M ratio was 1.88-fold higher in one-year-old beetles than in the current-year generation. The F/M ratio on shrubs was not dependent on light conditions, but the light × age interaction was significant. The F/M ratio was the highest in the one-year-old generation of beetles on shrubs growing in full light (Tukey’s HSD test; *P*<0.05).

**Table 2:** Mean values (and standard error) of *Altica brevicollis coryletorum* sex ratio (female to male beetles) in relation to insect age and light conditions, and a summary of the ANOVA results.

Treatment		Number of shrubs				Sex ratio (F/M)	
Light	Age	Site 1	Site 2		Site 3		
Full	Current	10	10		10	1.019 (0.090)	
	1-year	9	10		10	2.687 (0.528)	
Shade	Current	10	10		10	1.279 (0.170)	
	1-year	10	10		10	1.640 (0.223)	
ANOVA		d.f.		Error		*F*	*P*
Light		1		107.1		1.9079	0.1701
Age		1		107.1		12.2796	**0.0007**
Light × age		1		107.1		5.1603	**0.0251**
Light × site		2		107.1		3.0055	0.0537
Age × site		2		107.1		0.2260	0.7981
Light × age × site		2		107.1		2.3172	0.1035

Data was obtained from beetles at three different research sites (Kórnik, Palędzie, and Tulce).

### Laboratory experiment

The following results were obtained from the laboratory experiments investigating the effect of light conditions of common hazel seedlings on the growth and development of larvae and adults of *A*. *brevicollis coryletorum*. Out of the initial 40 individuals used from either sunlit or shades shrubs, 27 of the insects obtained from 100% full light shrubs survived till the end of the experiment, compared to 20 insects obtained from the 15% full light shrubs. Light conditions of the common hazel seedlings were found to significantly influence larval, pupal and beetle masses (Table 3). In all the developmental stages, insects fed with leaves of seedlings grown under full light were heavier than those fed with leaves from shrubs growing under limited (15%) light. The effect of insect sex was also significant, since at each stage females were heavier. The light × sex interaction, however, was significant only for larval mass. The difference between females and males was much higher in larvae fed with leaves of shaded seedlings than in larvae fed with leaves from full light seedlings. Neither light conditions nor sex significantly affected the efficiency of conversion of ingested food (ECI) and total food eaten (TFE). Despite this, the time needed for pupation, as measured by the duration of larval development (DD), was significantly extended in insects grown on leaves obtained from the 15% light shrubs than in insects grown on leaves from the 100% light shrubs. DD was greatly and significantly affected by sex and by the light × sex interaction (females fed with leaves of shaded seedlings pupated eight days earlier). The time needed for pupation in males fed with leaves from shaded seedlings was markedly longer than in the other three experimental variants (males fed with leaves of sunlit seedlings and females fed leaves from either shaded or sunlit seedlings). Relative growth rate (RGR) of larvae fed with leaves of seedlings from full light was much higher than in larvae fed with leaves from shaded seedlings. The light × sex interaction was also significant, as male larvae fed with leaves from shaded conditions, having lower RGR values, needed more time for time for larval development prior to pupation.

**Table 3:** Mean values (and standard error) of larval, pupal and beetle masses, efficiency of conversion of ingested food (ECI), duration of larval development (DD), total food eaten (TFE), relative growth rate after 10 days (RGR) per light condition treatment, and a summary of the ANCOVA results.

Treatment	Number of individuals		Larval mass (mg)		Pupal mass (mg)		Beetle mass (mg)		ECI (g g^-1^*100%)		DD (day)		TFE (mg dry mass)		RGR (g g^-1^ day ^-1^)	
Light	Female	Male	Female	Male	Female	Male	Female	Male	Female	Male	Female	Male	Female	Male	Female	Male
Full	18	9	7.79 (0.23)	6.70 (0.22)	7.64 (0.25)	6.48 (0.29)	5.65 (0.17)	4.23 (0.44)	6.08 (0.41)	5.86 (0.63)	20.44 (0.53)	20.67 (1.05)	137.20 (8.88)	124.87 (14.31)	0.85 (0.06)	0.72 (0.13)
Shade	5	15	6.99 (0.46)	4.13 (0.26)	6.45 (0.42)	3.94 (0.23)	4.92 (0.34)	2.64 (0.14)	4.74 (0.44)	5.36 (0.71)	19.20 (0.80)	27.20 (1.01)	126.41 (21.45)	94.53 (11.14)	0.51 (0.09)	0.28 (0.04)
ANCOVA	d.f.		*F*	*P*	*F*	*P*	*F*	*P*	*F*	*P*	*F*	*P*	*F*	*P*	*F*	*P*
Light	1		38.5517	**<0.0001**	38.4728	**<0.0001**	18.2314	**0.0001**	2.4931	0.1219	11.1220	**0.0018**	2.1773	0.1475	19.5577	**<0.0001**
Sex	1		27.7336	**<0.0001**	22.8704	**<0.0001**	36.0597	**<0.0001**	0.6333	0.4306	9.1701	**0.0042**	2.0659	0.1580	16.9592	**0.0002**
Light × sex	1		4.1346	**0.0484**	2.1943	0.1460	1.6034	0.2124	1.0391	0.3139	9.3328	**0.0039**	0.4050	0.5280	4.7493	**0.0350**
Initial larva mass (covariate)	1		6.5902	**0.0139**	2.7512	0.1046	0.3842	0.5387	1.8480	0.1813	7.3348	**0.0097**	0.0083	0.9278	21.1553	**<0.0001**
d.f. error			42		42		42		42		42		42		42	

Initial larva mass was used as a covariate to adjust initial mass differences between insects.

### Leaf toughness and chemical analysis

Results indicated that light conditions significantly impacted the toughness of leaves eaten during larval feeding (Fig 2A). The leaf toughness of shade leaves was 33% lower than sunlit leaves. Throughout the period of larval feeding, leaves of shaded seedlings had, on average, a 7.8% higher water content (Fig 2B) and a 31.4% higher nitrogen content (Fig 2E) than similar leaves obtained from seedlings growing in full light. In contrast, during the first half of the duration of larval feeding, leaves from full-light seedlings had a significantly higher concentration of total non-structural carbohydrates than leaves from shaded plants (Fig 2C; on successive dates: *P* = 0.0001, *P* = 0.0005, *P* = 0.1610 and *P* = 0.1898). In regards to defense-related compounds, leaves growing in full light had a 4.7-fold greater concentration of condensed tannins and a (Fig 2D) 3.4-fold greater concentration of soluble phenols (Fig 2F) than what was present in leaves from shaded shrubs.

### Histochemical analysis and anatomical measurements

The data from the present study indicated that leaves of sunlit shrubs in the field and seedlings grown in full light had a statistically significant, 1.9-fold (Fig 3A) and 2.1-fold (Fig 3B) higher density of trichomes (number of trichomes/mm^2^ of surface area), respectively, than leaves of shaded shrubs and seedlings. Additionally, the veins of sunlit leaves had a 2.3-fold higher density of trichomes than the veins of shades leaves.

Similarly to the total number of trichomes, better light conditions of shrubs corresponded with a greater density of glandular trichomes with phenols. Leaf blades of shrubs growing in full light had a significantly, 2.2-fold higher density of glandular trichomes with phenols than leaves of shaded shrubs (Fig 3C). Leaf blades of seedlings grown in full light had a 1.5-fold higher density of those trichomes than those grown in reduced light (Fig 3D). On leaf veins of sunlit shrubs, the density of glandular trichomes with phenols was significantly, 1.4-fold higher than on those of shaded shrubs (Fig 3C).

### Jump length

**Fig 4:**
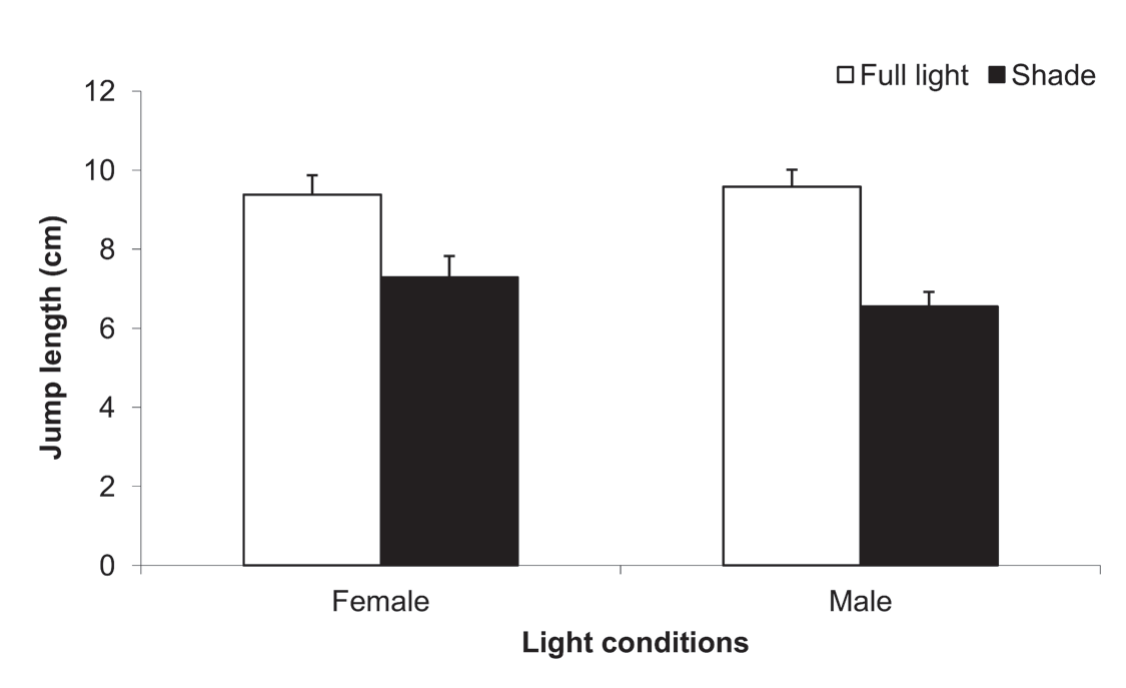
Jump length (cm) of *Altica brevicollis coryletorum* flea beetles obtained from leaves of shrubs growing under natural, sunlit or shaded light conditions in the field. One-way ANOVA was used to determine the significance of differences in jump length for light conditions (*P*<0.0001), sex (*P* = 0.9266) and light conditions × sex (*P* = 0.3161). Error bars denote standard error of the mean.

Results indicated that, under field conditions, beetle jump length was significantly impacted by whether the beetles resided on leaves of sunlit or shades shrubs (Fig 4). Insects obtained from leaves of sunlit shrubs jumped, on average, 1.38-fold further than those obtained from leaves of shaded shrubs. No significant effect of sex or a light × sex interaction on jump length was observed. Under laboratory conditions, the data indicated that temperature had a greater direct effect on the jump length of beetles than food quality (i.e. sunlit vs. shaded leaves, Table 4). The longest observed jump length was observed in beetles maintained at 35°C, whereas jump length in beetles kept at 15 and 25°C did not differ significantly (Tukey’s HSD test; *P*<0.05). Insect sex also did not have any significant effect on jump length. Lastly, no significant effect of beetle mass (as a covariate) on jump length was observed for the data obtained in the laboratory or the field.

**Table 4:** Mean values (and standard error) of *Altica brevicollis coryletorum* jump length, in relation to leaf type (light variant), temperature and sex, and a summary of the ANCOVA results.

Treatment		Number ofindividuals		Jump length (cm)	
Leaf type	Temperature	Female	Male	Female	Male
Full light	15°C	59	62	8.91 (0.39)	8.44 (0.32)
	25°C	41	65	8.56 (0.44)	8.98 (0.42)
	35°C	52	49	9.82 (0.40)	9.66 (0.40)
Shade	15°C	26	42	9.10 (0.52)	9.04 (0.39)
	25°C	51	48	10.69 (0.46)	10.36 (0.52)
	35°C	57	44	11.38 (0.46)	10.77 (0.62)
ANCOVA		d.f.	Error	*F*	*P*
Leaf type		1	1	0.0284	0.8663
Temperature		2	2	24.6385	**<0.0001**
Sex		1	1	0.0309	0.8606
Leaf type × Temperature		2	2	1.5198	0.2196
Leaf type × Sex		1	1	0.2446	0.6211
Temperature × Sex		2	2	0.2943	0.7451
Leaf type × Temperature × Sex		2	2	0.3853	0.6804
Beetle mass (covariate)		1	1	1.5925	0.2075

Beetle mass was used as covariate.

## Discussion

Results of this study indicate that leaves of *Corylus avellana* grown under sunlit conditions and used as a food source for larvae of the flea beetle, *Altica brevicollis coryletorum*, increase beetle mass. This applies to the results obtained from both field (Table 1) and laboratory studies (Table 3). In the field research, insects had the ability to choose their food source and were potentially impacted by other factors (variable temperature, competition between insects, predation, etc.), while in the laboratory study; insects could not choose food sources and environmental variables were largely controlled. The results of the laboratory experiment support the results obtained from the field research and indicate that the light conditions under which the common hazel shrub grows significantly affects insect mass in all developmental stages. Higher insect mass is the most frequently used indicator of better feeding conditions [33,34]. Higher food quality also leads to higher ECI and RGR for larvae [35,36]. Light did not affect ECI or TFE in our study, therefore, these parameters did not reveal which leaf type was better for insects. A higher nutritional value of leaves, however, does shorten DD and pupation time, which in turn decreases insect vulnerability due to attack by predators and parasites [36]. In the present study, higher level of light available to the sunlit shrubs caused remarkably, significant shortening of DD and an increase in RGR. Thus, the values of these parameters indicate that sunlit leaves possess a higher food quality which corresponds with the higher insect mass observed in all developmental stages for insects fed sunlit leaves.

Light conditions present during the time of growth of *C*. *avellana* seedlings corresponding with larval development (spring) had a significant impact on leaf toughness (Fig 2A). This, however, probably did not make them more difficult to eat since no significant effect of light conditions was observed on the amount of consumed food. Similarly, no significant effect of the level of nitrogen (N) on larval growth was observed, although shaded leaves were characterized by a significantly higher N content (Fig 2D). Nitrogen has a favorable impact on insect development [28,35,37], although it has been reasoned that the N content of plants is usually high and only extremely low values would affect insect development [38]. In contrast, carbohydrates in leaves of sunlit leaves act as specific feeding stimulants for insects [9,39]. The higher level of these constituents in sunlit leaves (Fig 2C) may explain the shortening of larval development and higher final mass (Table 3) for insects residing and feeding on sunlit leaves. Similar results were reported by Hwang and Lindroth [40] and Kaitaniemi et al. [41]. Therefore, we believe that the higher mass at all developmental stages, and the acceleration of larval development of *A*. *brevicollis coryletorum* observed in the present study, is caused primarily by the higher carbohydrate content of leaves of seedlings growing in full light. This may be explained by the fact that the increased carbohydrate content of leaves of sunlit shrubs attracts a larger number of insects of this species and consequently results in greater defoliation [9]. Thus our first hypothesis, that *A*. *brevicollis coryletorum* prefers sunlit leaves of *C*. *avellana* due to their greater nutritional value, primarily their higher non-structural carbohydrate content, was confirmed.

Most earlier studies have indicated that sunlit plants are less damaged by folivores because leaves of sunlit plants contain higher concentrations of repellents [42]. The high concentrations of defense compounds (soluble phenols and condensed tannins) in leaves of sunlit *C*. *avellana*, however, did not appear to hinder the occupation of the leaves or feeding by the folivore, *A*. *brevicollis coryletorum* (Fig 2D and 2F and [9]). This insect species is a typical example of monophagy, and such specialist insects seem to tolerate higher levels of repellents than generalist species [43]. Our results indicate that sunlit leaves of common hazel, relative to shaded leaves (15% of full sunlight), are characterized by higher levels of chemical repellents, greater leaf toughness (Fig 2A), and higher densities of all trichomes and glandular trichomes with phenols (Fig 3). The more rapid insect growth and development observed in insects reared on leaves obtained from common hazel seedlings grown in full light, and the higher adult beetle mass observed on leaves of sunlit shrubs in the field, however, confirm our second hypothesis that the greater toughness and density of trichomes on sunlit leaves are not factors that make their consumption difficult or deleterious to insect health.

Results of our field research indicate a significant effect of insect age on beetle mass (Table 1). Current-year generation beetles feeding in summer weighed less than those one-year-old beetles collected in spring. It should be noted, however that between the collection of insects in summer and the next catch in spring, beetles would have been feeding till late September. Leaf structure and chemistry is dependent on leaf age and it is assumed that young leaves are a better source of food for most insect folivores. Alonso and Herrera [44] reported that the nitrogen content of leaves declines with leaf age, while secondary metabolites (tannins) become more abundant. Older leaves are also tougher, more leathery, and therefore, theoretically more difficult for folivores to eat and digest [45]. This is why the higher insect mass recorded in the spring may be due to the higher nutritional value and easier consumption of young leaves. However, it may also be due, to the fact that, during the collection of beetles in the spring, some females may have already been fertilized. As previously mentioned, beetle mass did not differ in males. In spite of differences associated with beetle age, however, the relationships between beetle mass and the light conditions of shrub growth were similar.

Differences in F/M ratio, depending on insect age, were observed in our field study (Table 2). The ratio was higher in the one-year-old generation, which actively participates in reproduction, than it was in the current-year generation. The age × light interaction indicated that the F/M ratio was significantly higher on sunlit shrubs. The site of oviposition is greatly affected by the food preferences of females and the favorable potential of a site for offspring development [46,47]. Females lay eggs or produce offspring on the plant host that will result in the highest efficiency [47] and/or level of safety [48] for their offspring. Therefore, we conclude that the higher F/M ratio on sunlit shrubs indicates that females strive to ensure more favorable living conditions for themselves and the larvae of the next generation.

When analyzing the influence of light levels available to host plants on the feeding sites of folivores, both the direct effect and indirect effect (through food) of temperature should be taken into account. Temperature, beside nutrients and defense compounds, is one the major environmental factors affecting folivore growth and development [45]. Increasing temperature accelerates insect metabolism, which leads to an increase in insect activity, and thus the rate of development of eggs, larvae, and pupae [21,39,49]. Based on the collective data obtained in the present study, we conclude that in full sunlight, the higher temperature and higher level of non-structural carbohydrates available to *A*. *brevicollis coryletorum* provides more favorable conditions for growth and development, which is reflected by the fact that the highest adult insect mass was obtained in leaves and shrubs that were provided full light. Additionally, adult beetles of this species escape from threats more effectively at higher temperatures by jumping longer distances. These findings allow us to confirm the third hypothesis that the preference of beetles for sunlit shrubs provides a more effective defense against predators, partially due to the direct effect of temperature.

In general, results of the laboratory and field research are consistent with each other. They indicate that sunlit leaves of *C*. *avellana* are a more favorable food source for larvae of *A*. *brevicollis coryletorum* than shaded leaves. Sunlit leaves, relative to shade leaves, are characterized by greater thickness, density of trichomes and a higher level of defense compounds. Despite the higher levels of defense-related parameters in sunlit leaves, the growth and development of insects feeding on sunlit leaves is better than in shade leaves. The major reason for the higher beetle mass on sunlit shrubs seems to be due to the higher concentration of non-structural carbohydrates in sunlit leaves, relative to shade leaves. Moreover, as a specialist species, *A*. *brevicollis coryletorum*, during its co-evolution with *C*. *avellana*, certainly developed adaptations to overcome the defense of its host [50]. Thus, despite the higher concentration of phenolic compounds and trichromes density, the carbohydrate-rich sunlit leaves and higher temperature have a significant positive effect on the metabolism of this insect species and simultaneously increases the defense potential of the adult beetle. Among the insects found on *C*. *avellana*, only *A*. *brevicollis coryletorum* is a monophagy [6]. The flea beetle as monophagous species has specific relationship with its host plant. It means that it has adapted to high level of hosts defense compounds. It is assumed that this increased resistance results from a better adaptation of oligo- and monophagous than polyphagous insects in evolution and co-evolution of host plants.
